# Modern Aspects of Appendicitis. II

**Published:** 1893-12-09

**Authors:** 


### Modern Aspects of Appendicitis.—II.

Passing on from simple appendicitis, which we discussed in our last article, the next variety mentioned by Kelynack is perforative appendicitis, and this is clinically by far the most important. We quite agree with the author that the best practical classification is into (1) Perforative appendicitis with diffuse peritonitis; (2) perforative appendicitis with localized peritonitis; (3) perforative appendicitis with extra-peritoneal suppuration. It is a remarkable fact that perforative appendicitis is far more common in females than in males. Different statistics give from eleven to twenty-six per cent of the cases as occurring in males. At present there is no adequate explanation for this very marked difference. Another interesting fact in connection with this malady is that it is for the most part a disease of early life, most of the cases occurring between the ages of ten and thirty, whilst it is rarely met with after forty. There is no perfectly satisfactory reason for this; perhaps it is in part due to the fact that lymphoid tissue is very abundant in the appendix, and it is well known that lymphoid tissue is more prone to inflame in the earlier years of life than in the later. Kelynack mentions that he has found appendicitis to to be actually less common in lunatics than ordinary persons, which is important as bearing out the truth of the assertion that foreign bodies are not often the cause of it, for it is well known that insane persons are fond of swallowing foreign bodies. Traumatism is not of much value as a cause, except that it may convert a mild into a dangerous case. Certain micro-organism are constant inhabitants of the appendix; in the healthy state they are probably harmless.

The first form of perforative appendicitis to which our attention is directed is that which gives rise to diffuse peritonitis. Naturally there are two stages of this; first there must be gangrene of some part of the appendix, and then perforation will follow, and consequent upon the extension of the intestinal contents into the peritoneal cavity an acute diffuse peritonitis is set up. When this occurs the prognosis is very grave, and death commonly happens between the fifth and eighth day. Perhaps Kelynack expresses the surgical view rather too strongly, when he says, "Unless arrested by surgical interference its termination is almost invariably fatal." At any rate the cases he quotes do not tempt the physician to hand his patients over to the operating surgeon, for all the five cases he gives died—both those which were operated upon and those which were not. The great difficulty lies in the diagnosis; no doubt if that could always be made with certainty the right treatment would be to operate when undoubted suppurative peritonitis had occurred, but often less serious conditions, which would get perfectly well without operation, present symptoms indistinguishable from those of acute suppurative peritonitis.

The next class of case is headed perforative appendicitis with localised peritonitis, and "in very many instances of perforative appendicitis only a localised abscess results." In any case in which the diagnosis is made with reasonable certainty an operation should at once be undertaken, but unfortunately here also the difficulties are numerous for there are many cases of localised peritonitis due to appendicitis which never suppurate, and they, of course, are just the cases that get well without operation. In many of them there is a perforation; we have seen a case in point; in others probably there is no perforation. As showing the difficulty, we remember a case in which a distinguished surgeon and physician met over a patient one evening; the surgeon was so sure there was a localised abscess, which might burst into the general peritoneal cavity at any time, that he said he must, in justice to his patient, operate at once. The physician reluctantly yielded. No abscess was found, only localised peritonitis. The case probably would have got quite well without operation. Many writers on appendicitis appear to forget that in this disease non-suppurative peritonitis is far more frequent than suppurative.

Kelynack gives a brief account of tubercular and typhoid appendicitis, as he says it is very surprising that the former is so rare, for it might well be thought that tubercle bacilli would flourish in the lymphoid tissue, which is so abundant in the appendix; but out of five hundred cases of tubercular ulceration of the intestine, the appendix was only affected in seventeen, and it is quite possible that in some of these seventeen cases the ulceration was non-tubercular. With regard to the complications of appendicitis, out of 124 cases of acute peritonitis, only 26 were due to perforation of a viscus, and out of these 26, in only seven was the appendix the seat of the perforation. This, of course, strongly supports the contention that very many cases of appendicitis do not lead to acute general peritonitis. Fistulous communications between the appendix and some viscus, or a localized appendicular abscess are fairly common. Either may lead to the cæcum, the bladder, the rectum, or still more commonly they may open externally. Rare complications are pylephlebitis and suppurative hepatitis.

Just as it is remarkable that tubercular ulceration was so seldom met with in the appendix, so it is strange that no case of new growth has been recorded in a series of nearly sixteen thousand cases of neoplasms. There are, however, a few cases on record of new growth of the vermiform appendix, but probably in none of them was it primary.

A short account of the clinical symptoms of appendicitis is given, and attention is especially drawn to the well-known fact that frequently the pain, although at first felt in the right iliac fossa is later referred to the left. Kelynack leaves it an open question whether "McBurney's point" (which is a point an inch and a-half to two inches from the right anterior superior spine along a line from it to the umbilicus), is especially the seat of tenderness in appendicitis. He is, however, certain that it does not mark the seat of the base of the appendix. A short account is given of the difficulties of diagnosis, from which it appears that the physician should be particularly on the look out for simple excessive cæcal distension, intestinal obstruction, and movable kidney. There is nothing that calls for comment in the chapter on treatment, except that we think opium might be more freely given than is advised.

